# Role of Keratinized Mucosa on the Risk of Peri‐Implant Diseases and Soft Tissue Dehiscence in the Posterior Mandible—A 20‐Year Prospective Cohort Study

**DOI:** 10.1111/jre.70018

**Published:** 2025-07-21

**Authors:** Andrea Roccuzzo, Jean‐Claude Imber, Alexandra Stähli, Mario Romandini, Anton Sculean, Giovanni E. Salvi, Mario Roccuzzo

**Affiliations:** ^1^ Shanghai Perio‐Implant Innovation Center, Institute of Integrated Oral, Craniofacial and Sensory Research, Shanghai Ninth People's Hospital, Shanghai Jiao Tong University School of Medicine Shanghai China; ^2^ College of Stomatology, Shanghai Jiao Tong University, National Center of Stomatology, National Clinical Research Center for Oral Diseases, Shanghai Key Laboratory of Stomatology Shanghai China; ^3^ Department of Periodontology School of Dental Medicine, University of Bern Bern Switzerland; ^4^ Division of Maxillofacial Surgery University of Torino Torino Italy; ^5^ Department of Periodontics and Oral Medicine University of Michigan Ann Arbor Michigan USA; ^6^ Private Practice Torino Italy

**Keywords:** clinical trial, dental implants, free gingival graft, long‐term, soft tissue graft

## Abstract

**Aim:**

To evaluate the 20‐year outcomes of tissue‐level implants placed in the posterior mandible, comparing implants surrounded by keratinized tissue (KT) or alveolar mucosa (AM).

**Methods:**

At baseline, 128 patients (128 implants) were rehabilitated with implant‐supported fixed dental prostheses in the posterior mandible and enrolled in a supportive periodontal/peri‐implant care (SPC) program. Patients were categorized based on the presence (KT) or absence (AM) of keratinized mucosa. During the first 10 years of SPC, 11 AM patients underwent free gingival grafting (FGG), identifying a third group (AM + FGG). At the 20‐year follow‐up, peri‐implant health status and soft‐tissue dehiscence were assessed according to the 2018 Case Definitions. The need for additional treatment between the 10‐ and 20‐year examinations was also recorded.

**Results:**

Of the 98 patients evaluated at the 10‐year follow‐up, 64 (KT = 42; AM = 16; AM + FGG = 6; drop‐out rate: 35%) attended the 20‐year examination. Additional treatment was required in 11 AM patients (50%) versus 2 KT patients (5%) (*p* < 0.01). AM implants exhibited significantly greater marginal bone loss, bleeding on probing, and soft tissue recession compared to KT implants (*p* < 0.01). The application of an FGG (AM + FGG = 6) had a protective effect on peri‐implant health status at 20 years. Peri‐implantitis was diagnosed in 4.2% of implants surrounded by keratinized mucosa (KT or AM + FGG) versus 25% in the AM group (OR = 6.67; 95% CI: 1.09–40.9; *p* = 0.041).

**Conclusion:**

Tissue level implants placed in the posterior mandible without KT showed greater marginal bone loss, bleeding on probing, soft tissue recession, and peri‐implant diseases compared to implants with KT at 20 years.

## Graphical abstract

The application of an FGG at tissue level implants in the posterior mandible had a positive effect on clinical and radiographic outcomes and peri‐implant conditions after 20 years of function.

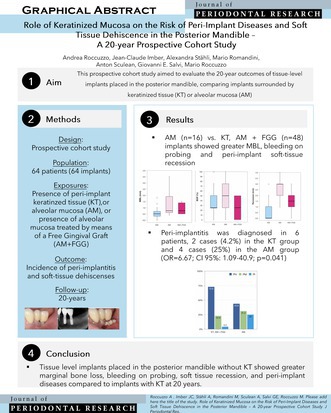

**Summary**

- Background
  ○ The potential protective role of peri‐implant keratinized mucosa on peri‐implant health and soft‐tissue dehiscence is still a controversial clinical topic.
- Added value of this study
  ○ The application of an FGG at tissue level implants in the posterior mandible had a positive effect on clinical and radiographic outcomes after 20–years of function.
- Clinical implications
  ○ The outcomes of the present long‐term study highlight the importance of the application of an FGG at implants in the posterior mandible lacking keratinized mucosa to maintain peri‐implant health and prevent soft‐tissue dehiscence.

## 1 Introduction

Dental implants have exceptionally improved oral rehabilitation of partially edentulous patients, being recognized as a highly predictable and reproducible treatment modality [1, 2, 3]. Historically, the most common reported outcomes to assess the quality of implant rehabilitation were mainly focused on the “hard tissues” and included implant survival rate and peri‐implant marginal bone level changes [4]. However, during the last two decades, with the introduction of esthetic parameters—which per se include an evaluation of the peri‐implant “soft‐tissues” conditions—and the adoption of the 2018 Case Definitions, the scientific community has shifted toward a more detailed assessment of the peri‐implant health status [5, 6].

In this context, the importance of peri‐implant soft tissues as risk indicators for peri‐implant diseases and soft‐tissue dehiscence is reflected in the number of recent systematic reviews [7, 8, 9] and clinical studies [10, 11, 12, 13, 14, 15, 16, 17]. More specifically, some studies suggested that implants lacking keratinized tissue (KT) are more prone to plaque accumulation, mucosa recession, and increased peri‐implant probing pocket depths (PPD) [18]. However, as indicated by a recent systematic review, additional studies with a longitudinal design and a long‐term follow‐up data are needed [19]. From a clinical perspective, this aspect is extremely relevant since peri‐implant soft‐tissue conditions are often modified through free gingival grafting (FGG). However, at the time being, no study ever documented the clinical and radiographic outcomes around implants with/without peri‐implant KT at 20 years. Considering increasing patients' life expectancy, it seems of paramount clinical relevance to monitor peri‐implant conditions in the long term.

Hence, the aim of this prospective cohort study was to report the 20‐year clinical and radiographic outcomes of tissue level implants placed in the posterior mandible in the presence or absence of peri‐implant KT in patients enrolled in an individualized supportive periodontal/peri‐implant care (SPC) program.

## 2 Methods

This manuscript adheres to the STROBE guidelines [20]. The study was conducted according to the Helsinki Declaration, as revised 2018, and all participants signed a written informed consent. The 20‐year study protocol was submitted to and approved by the Institutional Ethics Committee (Nr.168/2021).

### 2.1 Study Population

The original cohort consisted of 128 patients (128 implants) rehabilitated with sandblasted and acid‐etched surface (SLA) dental implants (Straumann Group AG, Basel, Switzerland). Details of the surgical and prosthetic procedures have been previously described [18]. Briefly, 128 patients (mean age 52 years; 16% smokers), attending the senior investigator's specialist periodontal practice in Torino, Italy, between December 1998 and December 2002 and seeking dental implant rehabilitation were included. From the original cohort, only subjects participating (*n* = 64) in both follow‐up visits (i.e., 10 and 20 years) were included in the present analysis.

The following exclusion criteria were applied at baseline:

- mucosal diseases
- alcohol and drug abuse
- pregnancy or breast feeding
- uncontrolled metabolic disorders
- severe chronic or aggressive periodontitis, according to Armitage 1999^21^
- inability or unwillingness to give informed consent.
- implants placed in previously regenerated bone

The following inclusion criteria were applied:

- Need of one implant in the posterior mandible (i.e., premolar/M) as a distal element;
- Implants placed in pristine bone with no need for guided bone regeneration (GBR) concomitant to implant placement.

Included patients were categorized as periodontally healthy (i.e., PHP—patient without signs of chronic periodontitis) or periodontally compromised patients (i.e., PCP—patients with a diagnosis of mild/moderate chronic periodontitis) following the case definitions by Armitage et al. [21].

### 2.2 Periodontal Therapy, Implant Therapy and Prosthetic Phase

After enrollment, all patients received appropriate initial therapy, consisting, depending on the case, of motivation, oral hygiene instructions, and periodontal non‐surgical/surgical therapy. No implant surgery was performed before optimal motivation and compliance from each patient were achieved (i.e., full mouth plaque score (FMPS) ≤ 20% and full mouth bleeding score (FMBS) ≤ 20%). Tissue‐level SLA implants were placed, under local anesthesia, by an experienced operator (MRoc), according to the manufacturer's instructions and using a standardized surgical procedure [22]. No soft tissue augmentation procedures were performed either prior to or concomitant with implant surgery. Abutment connection was carried out after 6–10 weeks of non‐submerged healing, and all patients were provided with cemented implant‐supported fixed metal‐ceramic restorations. After restoration delivery (T0), patients were enrolled in an individualized Supportive Periodontal and Peri‐implant Care (SPC) program. Specifically, they were regularly recalled for oral hygiene instructions, supra‐marginal instrumentation, and treatment of peri‐implant diseases, if needed. SPC was performed by an experienced dental hygienist.

### 2.3 Keratinized Mucosa Assessment

The presence (KT) or absence (AM) of keratinized mucosa was visually assessed at T0 at the mid‐buccal aspect of each study implant by an experienced operator (M.Roc). Within the first 10 years of SPC, whenever AM patients did not show proper plaque control at the study implant and this condition was associated with self‐reported soreness during oral hygiene procedures, they were given the option to receive a free gingival graft (AM + FGG). No additional soft tissue grafting procedures were performed between 10 and 20 years. This led to the identification of 3 groups as the exposure: KT, AM, and AM + FGG.

### 2.4 Clinical and Radiographic Outcomes

A patient not attending all follow‐up examinations was classified as “drop‐out”. At the 10‐year (T1) and 20‐year (T2) follow‐up examination, implant survival (i.e., presence of the implant in the oral cavity) was recorded. Moreover, the same experienced dental hygienist, blinded to the scopes of the study, recorded PPD (mm) measured at four sites (mesial, buccal, distal, and lingual) by means of a graduated periodontal probe (XP23/UNC 15; Hu‐Friedy). Measurements were rounded off to the nearest millimeter. Presence of plaque at the implant level (Pl), bleeding on probing (BOP), and suppuration was additionally recorded. Soft‐tissue recession was also measured from the implant shoulder to the coronal mucosal margin, by means of a Castroviejo Caliper Short (Salvin Dental Specialties Inc., Charlotte, NC, USA) and rounded to the nearest ½ millimeter [23].

At T1 and T2 examinations, the following parameters were recorded at patient level:

- FMPS, as the percentage of sites exhibiting Pl measured at four sites per tooth/implant;
- FMBS, as the percentage of sites exhibiting BOP measured at four sites per tooth/implant;
- Adherence to SPC (yes or no), defined as full compliance with the SPC program proposed by the principal investigator taking into account the patient's needs and risk profile;
- History of either C or D CIST (Cumulative Interceptive Supportive Therapy) modalities (i.e., antibiotics and/or surgery) [24] during SPC.

A periapical radiographic image was obtained to assess peri‐implant marginal bone level changes, following a methodology previously proposed [25] and validated [26]. Specifically, non‐standardized periapical radiographs were collected using the long‐cone technique. Each image was then calibrated based on the known implant length and analyzed using a dedicated software (ImageJ, National Institutes of Health, Bethesda, MD, USA). The distance between the base of the implant shoulder and the most coronal visible bone‐to‐implant contact was measured in millimeters at both the mesial and distal aspects of each implant, with the highest value recorded. Mean marginal bone loss (MBL) was calculated by subtracting the 20‐year bone level from the baseline (T0) value. All radiographies were assessed in duplicate by two experienced, previously calibrated examiners (J‐C.I, A.St.) who were not involved in treatment or follow‐up examinations (Kappa score = 0.906; 95% CI [0.893; 0.998]; *p* < 0.001). Finally, peri‐implant health status at 20 years (i.e., health (PH), peri‐implant mucositis (PM) and peri‐implantitis (PI)) was determined according to Renvert et al. 2018 [27], while soft‐tissue dehiscence at the implant site was defined as REC ≥ 1 mm.

### 2.5 Data Analysis

All analyses were performed using an ad hoc statistical software (STATA BE, version 17.1, StataCorp, Texas, USA), setting the significance level at 5% (alfa = 0.05). Patients and implant variables were described using mean, standard deviation, range, median, and interquartile range for continuous and absolute and relative frequencies for categorical ones. Shapiro–Wilk's test detected non‐normal distributions and consequently a non‐parametric approach was considered for the inferential analysis. Chi2 and Fisher's exact tests were used to assess associations between categorical variables. Mann–Whitney's and Kruskal–Wallis tests were applied to compare distributions of ordinal/continuous variables between groups (i.e., two or three respectively). Correlations were estimated using Spearman's coefficient. Univariable and multivariable binary logistic regressions providing crude and adjusted OR were estimated to study whether the presence or absence of KT at baseline was associated with peri‐implant health status at 20 years.

## 3 Results

Of the 98 patients evaluated at the 10‐year follow‐up, 34 were lost at the 20‐year follow‐up, resulting in an attrition rate of 35%. Specifically, 11 patients had passed away, while 23 declined to attend the follow‐up visit. The number of patients lost to follow‐up over the 20‐year period is reported in Table 1. No implants were lost between the 10‐ and 20‐year examinations. Thus, the cohort analyzed at the 20‐year follow‐up included 64 subjects (64 implants) (Table 1).

**TABLE 1:** Number of patients (implants) over the 20‐year study period; number of implants examined and reasons for drop‐out.

	Patients	Patients lost to follow‐up
Baseline	128	—
10‐year	98	30
20‐year	64	34
*Reasons for drop‐out between 10 and 20 years*		
Death	11	
Refused to attend a follow‐up visit	23	
Total	34	

Patients' characteristics, stratified by implant placement in keratinized tissue (KT = 42) or alveolar mucosa (AM = 22), are summarized in Table 2. There were no significant differences between groups in terms of gender, age, smoking status, adherence to SPC, and periodontal status (*p* > 0.05). However, AM patients exhibited a significantly higher FMBS score compared to KT patients (21.8% vs. 18.8%, *p* = 0.024).

**TABLE 2:** Patients' characteristics at the 20‐year follow‐up with respect to the originally classification of the test implant placed in keratinized tissue (KT) vs. alveolar mucosa (AM).

	KT (*n* = 42)	AM (*n* = 22)	*p*
Male gender	23 (54.8%)	13 (59.1%)	0.740 (Chi^2^)
Age at baseline	50.7 ± 9.7	52.4 ± 8.3	0.424 (MW)
Smoking	6 (14.3%)	0 (0%)	0.086 (Fis)
Compliance with SPC	36 (85.7%)	18 (81.8%)	0.683 (Chi^2^)
mPCP	26 (61.9%)	14 (63.6%)	0.892 (Chi^2^)
CIST C/D	2 (4.8%)	11 (50.0%)	**0.001 (Chi** ^**2**^)
*FMPS (%)*			
Mean ± SD	19.6 ± 9.1	20.4 ± 6.7	0.250 (MW)
Median (25–75)	18 (12–23)	21 (15–26)	
*FMBS (%)*			
Mean ± SD	18.8 ± 6.0	21.8 ± 6.1	**0.024 (MW)**
Median (25–75)	17.5 (15–22)	22 (18–25)	
PH/PM/PI	30/8/2 (75%/20%/5%)	11/7/4 (50%/31.8%/18.2%)	0.095 (Chi^2^)
PH/PM + PI	30/10 (75%/25%)	11/11 (50%/50%)	**0.047 (Chi** ^**2**^)

*Note:* Results (*p*‐value) of Chi^2^, Fisher's exact test (Fis) or Mann–Whitney's test (MW). Bold values correspond to statistically significant difference.

Abbreviations: AM, alveolar mucosa; CIST, cumulative interceptive supportive therapy; FMBS, full mouth bleeding Score; FMPS, full mouth plaque score; KT, keratinized tissue; mPCP, moderate periodontally compromised patients; PH, peri‐implant health; PI, peri‐implantitis; PM, peri‐implant mucositis; SD, standard deviation; SPC, supportive periodontal/peri‐implant care.

### 3.1 Peri‐Implant Clinical and Radiographic Parameters at 20‐Year Follow‐Up

The 20‐year clinical and radiographic outcomes for the three groups are summarized in Table 3. No statistically significant differences were observed in PI (*p* = 0.623) and PPD (*p* = 0.848), although a trend toward higher PPD values was noted in the AM group (3.56 mm) compared to KT (2.95 mm) and AM + FGG (2.83 mm).

**TABLE 3:** Parameters at 20‐year follow‐up around the implants according to their status: Originally placed in keratinized tissue (KT), in alveolar mucosa (AM), and in alveolar mucosa with additional FGG (AM + FGG).

	KT (*n* = 42)	AM (*n* = 16)	AM + FGG (*n* = 6)	*p* (KW)	KT vs. AM	*p* (MW) KT vs. AM + FGG	AM vs. AM + FGG
mBL (mm)							
Mean ± SD	0.66 ± 0.70	1.29 ± 0.99	0.90 ± 0.44	**0.004**	**0.006**	0.201	1.000
Median (25–75)	0.5 (0.3–0.7)	0.75 (0.6–1.9)	0.75 (0.5–1.4)				
PPD (mm)							
Mean ± SD	2.95 ± 0.92	3.56 ± 1.73	2.83 ± 0.34	0.848	1.000	1.000	1.000
Median (25–75)	2.75 (2.5–3.0)	2.88 (2.5–4.5)	2.75 (2.5–3.2)				
BOP (%)							
Mean ± SD	30.9 ± 23.9	51.6 ± 26.6	25.0 ± 22.4	**0.012**	**0.015**	1.000	0.177
Median (25–75)	25 (25–50)	50 (25–75)	25 (0–50)				
PII (%)							
Mean ± SD	29.2 ± 22.7	34.4 ± 22.1	33.3 ± 30.3	0.623	1.000	1.000	1.000
Median (25–75)	25 (25–50)	37.5 (25–50)	37.5 (0–50)				
REC (mm)							
Mean ± SD	0.43 ± 0.77	2.69 ± 0.79	1.50 ± 1.05	**< 0.001**	**< 0.001**	**0.048**	0.081
Median (25–75)	0 (0–1)	3 (2–3)	1.5 (1–2)				
PH/PM/PI	31/9/2 75%/20%/5%	7/5/4 43.8%/31.1%/25%	4/2/0 66.7%/33.3%/0%	0.056	**0.018**	0.843	0.294
PH/PM + PI	31/11 75%/25%	7/9 43.8%/56.2%	4/2 66.7%/33.3%	0.102	0.031	0.796	0.449

*Note:* Results (*p*‐value) of Kruskal Wallis (KW) and Mann–Whitney's test (MW) with Bonferroni's correction. Bold values correspond to statistically significant difference.

Abbreviations: AM, alveolar mucosa; BOP, presence of bleeding on probing around the implant (%); FGG, free gingival graft; KT, keratinized tissue; mBL, mean marginal bone loss (mm); PH, peri‐implant health; PI, peri‐implantitis; PII, plaque index at implant level (%); PM, peri‐implant mucositis; PPD, Probing pocket depth (mm); REC, soft‐tissue recession (mm); SD, standard deviation.

**FIGURE 1:**
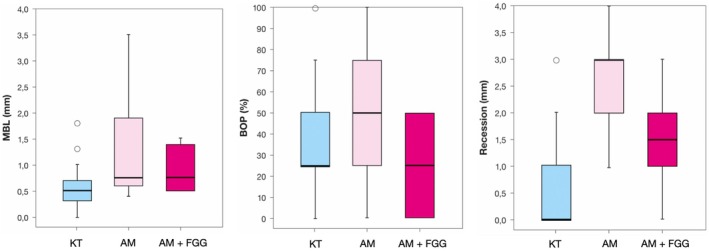
Box plots describing mean differences among the three groups (KT, keratinized tissue; AM, alveolar mucosa; AM + FGG, alveolar mucosa + free gingival graft) at the 20‐year examination with respect to the variables mean bone loss (MBL), bleeding on probing (BOP), and mucosal recession (REC).

MBL was highest in the AM group (i.e., 1.29 ± 0.99), followed by AM + FGG (i.e., 0.90 ± 0.44) and KT implants (i.e., 0.66 ± 0.70), with statistically significant differences among groups (*p* = 0.004) (Figure 1). A similar trend was observed for BOP and REC, with AM implants displaying the highest values: 52% (*p* = 0.012) and 2.9 mm (*p* = < 0.001), respectively. Throughout the follow‐up period, AM implants required more frequent intervention according to the CIST protocol (i.e., 11 vs. 2) (*p* = 0.001) compared to KT implants. Table 4 summarizes the changes between the 10‐ and 20‐year examination, indicating consistent results (for MBL values at the 10‐year follow‐up see Table S1).

**TABLE 4:** Clinical parameters changes (i.e., differences) between the 10 and 20‐year follow‐up around the test implants according to their status: Originally placed in keratinized tissue (KT), in alveolar mucosa (AM), and in alveolar mucosa with additional FGG (AM + FGG).

	KT (*n* = 42)	AM (*n* = 16)	AM + FGG (*n* = 6)	*p* (KW)	KT vs. AM	*p* (MW) KT vs. AM + FGG	AM vs. AM + FGG
Diff. mBL (mm)							
Mean ± SD	0.32 ± 0.56	0.84 ± 0.80	0.28 ± 0.17	**< 0.001**	**< 0.001**	1.000	0.081
Median (25–75)	0.2 (0.1–0.3)	0.5 (0.3–1.2)	0.28 (0.2–0.4)				
Diff. PPD (mm)							
Mean ± SD	−0.14 ± 0.83	0.78 ± 1.68	0.04 ± 0.97	0.153	0.165	1.000	1.000
Median (25–75)	−0.25 (−0.75 to 0.25)	0.25 (−0.5 to 2)	0.13 (−0.75 to 0.5)				
Diff. BOP (%)							
Mean ± SD	5.95 ± 23.3	21.9 ± 28.7	4.17 ± 10.2	**0.024**	**0.027**	1.000	0.462
Median (25–75)	0 (0 0)	25 (0 25)	0 (0 0)				
Diff. PII (%)							
Mean ± SD	6.55 ± 18.4	3.13 ± 17.9	12.5 ± 13.7	0.328	1.000	1.000	0.693
Median (25–75)	0 (0 25)	0 (0 0)	12.5 (0 25)				
Diff. REC (mm)							
Mean ± SD	0.20 ± 0.58	0.59 ± 0.49	0.17 ± 0.41	**0.002**	**0.003**	1.000	0.345
Median (25–75)	0 (0–0)	1 (0–1)	0 (0–0)				

*Note:* Results (*p*‐value) of Kruskal Wallis (KW) and Mann–Whitney's test (MW) with Bonferroni's correction. Bold values correspond to statistically significant difference.

Abbreviations: AM, alveolar mucosa; BOP, presence of bleeding on probing around the implant (%); Diff., difference; FGG, free gingival graft; KT, keratinized tissue; mBL, mean marginal bone loss (mm); PII, plaque index at implant level (%); PPD, Probing pocket depth (mm); REC, soft‐tissue recession (mm); SD, standard deviation.

### 3.2 Peri‐Implant Diseases at the 20‐Year Follow‐Up

**FIGURE 2:**
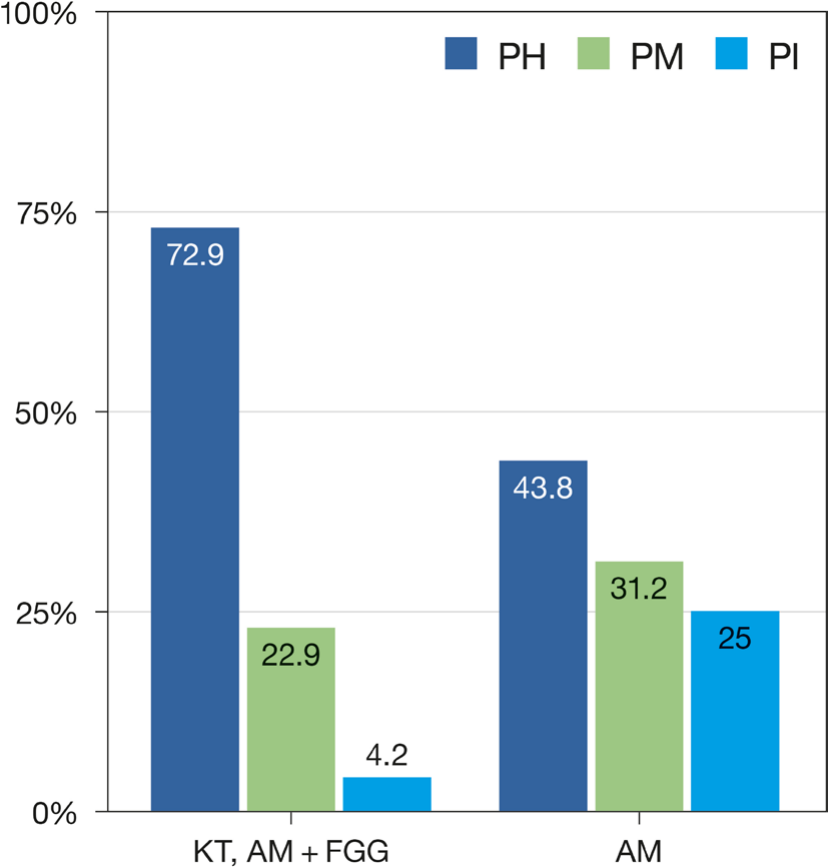
Peri‐implant conditions (i.e., peri‐implant health (PH), peri‐implant mucositis (PM) and peri‐implantitis (PI)) at the 20‐year examination with respect to the presence of keratinized tissue (i.e., KT, AM + FGG) or alveolar mucosa (AM).

Peri‐implant health status at the 20‐year follow‐up is summarized in Table 5. Peri‐implant health was diagnosed in 35 patients/implants (72.9%) in the KT and AM + FGG groups, compared to 7 patients (43.8%) in the AM group. Peri‐implant mucositis was observed in 11 cases (22.9%) in the KT and AM + FGG groups and 5 (31.2%) in the AM group. Peri‐implantitis was diagnosed in 6 patients: 2 cases (4.2%) in the KT group and 4 cases (25%) in the AM group (OR = 6.67; 95% CI: 1.09–40.9; *p* = 0.041). The difference among the three groups was statistically significant (*p* = 0.017) (Figure 2). Comparable trends were observed when grouping peri‐implant mucositis with either peri‐implantitis (*p* = 0.014) or peri‐implant health (*p* = 0.035) (for peri‐implant conditions at the 10‐year follow‐up see Table S2). Both the ordinal and binary logistic regression models revealed a statistically significant increased risk for peri‐implantitis (OR 5.15; 95% CI = 1.52–17.5; *p* = 0.009 & OR 19.1; 95% CI = 1.72–211.9; *p* = 0.016, respectively) for the AM group compared to the KT and AM + FGG groups (Tables S3 and S4).

**TABLE 5:** Peri‐implant conditions at the 20‐year follow‐up around the test implants according to their status: Originally placed in keratinized tissue (KT) and alveolar mucosa with additional FGG (AM + FGG) vs. in alveolar mucosa (AM).

	KT, AM + FGG (*n* = 48)	AM (*n* = 16)	*p* (MW)
PH/PM/PI	35/11/2 72.9%/22.9%/4.2%	7/5/4 43.8%/31.2%/25%	**0.017**
PH + PM/PI	46/2 95.8%/4.2%	12/4 75%/25%	**0.014**
PH/PM + PI	35/13 72.9%/27.1%	7/9 43.8%/56.2%	**0.035**

*Note:* Results (*p*‐value) of Mann–Whitney's test (MW). Bold values correspond to statistically significant difference.

Abbreviations: AM, alveolar mucosa; FGG, free gingival graft; KT, keratinized tissue; PH, peri‐implant health; PI, peri‐implantitis; PM, peri‐implant mucositis.

Soft‐tissue dehiscence was detected around all 16 AM implants (100%), while around only 17 implants (35.4%) with keratinized tissue (KT/AM + FGG): this difference was statistically significant (*p* < 0.001) (Table 6). Finally, AM implants showed a higher probability of soft‐tissue dehiscence than KT ones (OR 81.6; 95% CI = 8.45785.9; *p* < 0.001) (Table S5).

**TABLE 6:** Presence of soft‐tissue dehiscence (i.e., REC ≥ 1 mm) at the 20‐years follow‐up around the test implants according to their status: Originally placed in keratinized tissue (KT) and alveolar mucosa with additional FGG (AM + FGG) vs. in alveolar mucosa (AM).

	KT, AM + FGG (*n* = 48)	AM (*n* = 16)	*p* (Chi^2^)
0 mm ≥ 1 mm	31 (64.6%) 17 (35.4%)	0 (0%) 16 (100%)	**< 0.001**

*Note:* Results (*p*‐value) of Chi^2^ test. Bold values correspond to statistically significant difference.

## 4 Discussion

The present study aimed to document the 20‐year clinical and radiographic outcomes at the tissue level for implants placed in the posterior mandible with respect to the presence (KT) or absence (AM) of keratinized mucosa and its impact on peri‐implant health. Based on the obtained results, it can be concluded that the presence of KT, whether present at the time of implant placement or grafted during SPC, had a significant beneficial effect.

Since the publication of the first 10‐year report on this cohort [18], peri‐implant soft tissue conditions have gathered scientific interest, as shown by the high number of systematic reviews published on this topic [28, 29, 30]. Recently, based on the results published by Stefanini et al. indicating that peri‐implant augmented sites maintain stable soft tissue margin and marginal bone levels at least after 3 years, while non‐augmented sites may display soft‐tissue apical margin shift [31], the application of FGG in posterior sites to facilitate patient's self‐plaque control as well as to reduce patient's discomfort was recommended [32]. One clinical question still open is related to the timing of such intervention [33]. Based on available evidence, a soft‐tissue augmentation procedure might be performed prior to implant placement in case of lack of KT or in case of bone augmentation procedures (i.e., horizontal/vertical GBR) to obtain optimal peri‐implant soft‐tissue conditions helping flap handling and post‐operative healing [34]. In the present study, six AM patients were treated with an additional graft, providing evidence that an accurate assessment followed by proper treatment of peri‐implant soft tissue defects should be encompassed within the SPC regimen.

The scientific rationale of the importance of performing peri‐implant soft‐tissue augmentation procedures has been recently provided in a pre‐clinical dog model. Following complete excision of KT around TL implants, spontaneous regeneration of the soft tissue was characterized by a non‐keratinized epithelium similar to AM, while at the control tooth sites the healing was characterized by keratinized soft tissue [35, 36]. Consequently, clinicians should consider peri‐implant soft‐tissue conditions assessment and treatment as a key factor of the implant‐supported rehabilitation.

From a clinical perspective, it is common experience, especially in the posterior mandible sites, that not only the quality of the peri‐implant soft‐tissue conditions (i.e., keratinized vs. non‐keratinized), but also the vestibular depth as well as the presence of “mobile” vs. “attached” mucosa play an important role in plaque accumulation and consequently on the eventual on‐set of peri‐implant diseases [9]. In the present study, these clinical variables were not systematically assessed and, therefore, additional conclusions cannot be drawn.

**FIGURE 3:**
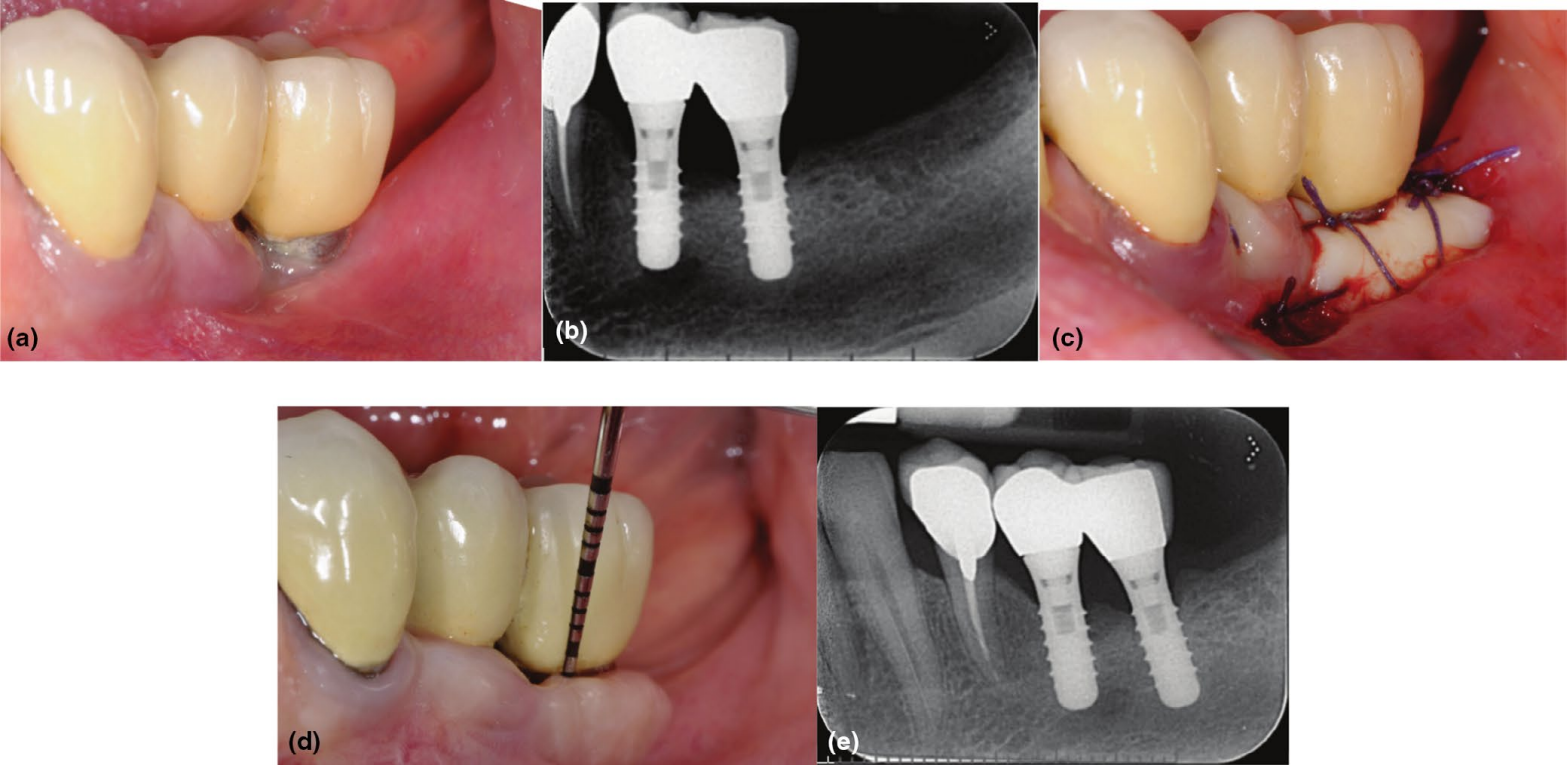
Clinical (a) and radiographic (b) views of the distal implant in region 3.6, placed 8 years earlier, demonstrate the lack of keratinized tissue (KT), peri‐implant soft‐tissue recession, and plaque accumulation. A free gingival graft (FGG) was performed to enhance the peri‐implant soft‐tissue conditions (c). Clinical (d) and radiographic (e) evaluations at the 20‐year follow‐up reveal minimal peri‐implant probing pocket depth, no bleeding on probing, and stable peri‐implant soft‐tissue conditions.

The assessment of peri‐implant health and disease has become a pivotal aspect within the documentation of the long‐term reliability of dental implants [37, 38, 39, 40]: in the present study, peri‐implant mucositis was detected around 22.9% KT versus 31.2% KT + AM/FGG implants. This difference was even greater when peri‐implantitis was found (4.2% vs. 25%) (Figure 3). These findings corroborate pre‐clinical data providing evidence of an increased susceptibility to the development of experimental peri‐implantitis in the absence of keratinized tissue [41].

When comparing our results with those available in the literature, they corroborate similar recent 10‐year data on 148 implants, where 20% of implants diagnosed with peri‐implantitis were associated with lack of KT (KT = 0 mm) [42]. One aspect that should be underlined since it might have affected the results is the cohort selection. Patients affected by severe periodontitis as well as heavy smokers were excluded and almost all included patients were compliant with a strictly tailored SPC program (i.e., > 80% compliant with the proposed SPC), as documented by the low mean FMBS and FMBS (i.e., < 25%). In addition, the implants examined in the present cohort were defined at baseline with or without peri‐implant keratinized mucosa, while those analyzed by Mancini and co‐workers were lacking such categorization, providing relevant but cross‐sectional evidence. In this context, however, it should be underlined that the evidence on the topic is still inconclusive, given that other prospective cohort studies failed to detect an association between KT and peri‐implantitis incidence [13]. Nevertheless, based on the present data, the protective effect of a FGG can be suggested.

Contemporary implant dentistry research should include patient's preferences and perspectives [1]. The present study design lacks assessment and reporting of specific patient's reported outcome measures (PROMs). It must be emphasized, however, that at the time of study conception (i.e., 1998), such topics were not of primary interest, especially in a private practice setting, and consequently, they were not specifically investigated.

This study retrieves several limitations. First, it must be underlined how, despite being prospective, the observational study design can expose participants to the risk of confounding bias. Second, the relatively high drop‐out rate (i.e., 35%) might have reduced statistical power for non‐significant estimates. However, this is an inherent issue with long‐term studies, as shown by other reports with comparable follow‐ups [43, 44] and that the analyzed implant system is still available on the market, consequently providing important valuable clinical information. Third, the assessment of the clinical measurements did not follow a calibration session, even though they were collected by the same experienced dental hygienist. In addition, with respect to the radiographic analysis, this was performed by means of periapical radiographs, as it is performed in daily clinical practice. Periapical radiographs, however, still represent the standard of care in long‐term monitoring of dental implants in a private practice setting and are to be considered adjunctive to the assessment of clinical peri‐implant parameters. Consequently, precise information on the peri‐implant bone morphology in the bucco‐lingual dimension is missing. Furthermore, the limited sample size of the AM + FGG group, with the consequent wide observed confidence intervals, should be disclosed. Finally, it should be underlined that the overall external validity of the presented data might be reduced since all treatments were performed by the same experienced clinician in a specialist setting including one dental implant system only (i.e., tissue level implant).

## 5 Conclusions

Tissue level implants placed in the posterior mandible without KT showed greater marginal bone loss, bleeding on probing, soft tissue recession, and peri‐implant diseases compared to implants with KT at 20 years.

## Author Contributions

A.R. contributed to study conception and collected the data; A.R. and J.‐C. I analyzed the data and led to the writing; A.St., G.E.S. analyzed the data and contributed to the writing; M.Rom. contributed to study conception and critically revised the manuscript; A.S., critically revised the manuscript; M.Roc. conceived the idea, performed the treatment and critically revised the manuscript.

## Conflicts of Interest

The authors declare no conflicts of interest.

## Supporting information


**Table S1.** 10‐year mean MBL by group (KT, AM + FGG, AM).
**Table S2.** Peri‐implant conditions at the 10‐years follow‐up around the test implants according to their status: originally placed in keratinized tissue (KT), in alveolar mucosa (AM), and in alveolar mucosa with additional FGG (AM + FGG).
**Table S3.** Ordinal logistic regression (crude and adjusted odds ratio OR; 95% CI) by group (KT, AM + FGG/AM) and other independent factors at the 20‐year follow‐up.
**Table S4.** Binary logistic regression (crude and adjusted odds ratio OR; 95% CI) by group (KT + AM + FGG/AM) and other independent factors at the 20‐year follow‐up.
**Table S5.** Presence of peri‐implant soft‐tissue dehiscence (i.e., REC > 1 mm). Binary logistic regression (crude and adjusted odds ratio OR; 95% CI) by group (KT/AM) and other independent factors at the 20‐year follow‐up.

## Data Availability

The data that support the findings of this study are not publicly available to privacy or ethical restrictions.
