# Supplementary material for: Role of Keratinized Mucosa on the Risk of Peri‐Implant Diseases and Soft Tissue Dehiscence in the Posterior Mandible—A 20‐Year Prospective Cohort Study
Source: J Periodontal Res. 2025 Jul 21;60(12):1212–21. doi: 10.1111/jre.70018 (PMC12881885; doi:10.1111/jre.70018)
Supplement: Supplementary file 1 — Table S1. 10‐year mean MBL by group (KT, AM + FGG, AM). Table S2. Peri‐implant conditions at the 10‐years follow‐up around the test implants according to their status: originally placed in keratinized tissue (KT), in alveolar mucosa (AM), and in alveolar mucosa with additional FGG (AM + FGG). Table S3. Ordinal logistic regression (crude and adjusted odds ratio OR; 95% CI) by group (KT, AM + FGG/AM) and other independent factors at the 20‐year follow‐up. Table S4. Binary logistic regression (crude and adjusted odds ratio OR; 95% CI) by group (KT + AM + FGG/AM) and other independent factors at the 20‐year follow‐up. Table S5. Presence of peri‐implant soft‐tissue dehiscence (i.e., REC > 1 mm). Binary logistic regression (crude and adjusted odds ratio OR; 95% CI) by group (KT/AM) and other independent factors at the 20‐year follow‐up. [file JRE-60-1212-s001.docx]

**Supplementary Table 1.** 10-year mean MBL by group (KT, AM + FGG, AM)

|  | **KT**  **(n=42)** | **AM**  **(n=16)** | **AM+FGG**  **(n=6)** | **p-value**  **(KW)** |  | **KT vs. AM** | **p-value (MW)**  **KT vs. AM+FGG** | **AM vs. AM+FGG** |
| --- | --- | --- | --- | --- | --- | --- | --- | --- |
| **MBL (mm)**  mean ± SD  median (25-75) | 0.34 ± 0.40  0.25 (0.0-0.50) | 0.44 ± 0.39  0.50 (0.0-0.75) | 0.63 ± 0.31  0.50 (0.50-1) | 0.110 |  | 0.915 | 0.162 | 0.882 |

Results (p-value) of Kruskal Wallis (KW) and Mann-Whitney´s test (MW) with Bonferroni´s correction

AM, alveolar mucosa; FGG, free gingival graft; KT, keratinized tissue; MBL, marginal bone loss.

**Supplementary Table 2.** Peri-implant conditions at the 10-years follow-up around the test implants according to their status: originally placed in keratinized tissue (KT), in alveolar mucosa (AM), and in alveolar mucosa with additional FGG (AM+FGG).

|  | **KT, AM+FGG**  **(n=48)** | **AM**  **(n=16)** | **p-value**  **(MW)** |
| --- | --- | --- | --- |
| **PH/PM/PI** | 33/15/0  68.8%/31.3%/0% | 7/9/0 43.8%/56.3%/0% | 0.076 |
| **PH+PM/PI** | 48/0  100%/0% | 16/0  100%/0% | 1 |
| **PH/PM+PI** | 33/15  68.8%/31.3% | 7/9  43.8%/56.3% | 0.076 |

Results (p-value) of Mann-Whitney’s test (MW)

AM, alveolar mucosa; FGG, free gingival graft; KT, keratinized tissue; PH, peri-implant health; PI, peri-implantitis; PM, peri-implant mucositis.

**Supplementary Table 3.** Ordinal logistic regression (crude and adjusted odds ratio OR; 95%CI) by group (KT, AM+FGG / AM) and other independent factors at the 20-year follow-up

|  | **Simple model** | | |  | **Multiple model** | | |
| --- | --- | --- | --- | --- | --- | --- | --- |
|  | **Crude OR** | **95% CI** | **p-value** |  | **Adjusted OR** | **95% CI** | **p-value** |
| **GROUP** |  |  |  |  |  |  |  |
| KT, AM+FGG | 1 |  |  |  | 1 |  |  |
| AM | 4.08 | 1.32 12.7 | **0.015** |  | 5.15 | 1.52 17.5 | **0.009** |
| **Gender** |  |  |  |  |  |  |  |
| Male | 1 |  |  |  | 1 |  |  |
| Female | 1.14 | 0.41 3.15 | 0.807 |  | 1.22 | 0.41 3.58 | 0.722 |
| **AGE** | 1.01 | 0.96 1.07 | 0.677 |  | 1.00 | 0.94 1.07 | 0.934 |
| **SMOKING** |  |  |  |  |  |  |  |
| No | 1 |  |  |  | 1 |  |  |
| Yes | 2.07 | 0.41 10.4 | 0.378 |  | 2.97 | 0.51 17.2 | 0.224 |
| **SPC** |  |  |  |  |  |  |  |
| No | 1 |  |  |  | 1 |  |  |
| Yes | 0.52 | 0.14 1.94 | 0.330 |  | 0.69 | 0.17 2.83 | 0.611 |
| **mPCP** |  |  |  |  |  |  |  |
| No | 1 |  |  |  | 1 |  |  |
| Yes | 1.57 | 0.53 4.62 | 0.411 |  | 1.74 | 0.54 5.60 | 0.356 |

AM, alveolar mucosa; FGG, free gingival graft; KT, keratinized tissue; mPCP, moderate periodontally compromised patients; SPC, supportive periodontal/peri-implant care.

**Supplementary Table 4.** Binary logistic regression (crude and adjusted odds ratio OR; 95%CI) by group (KT + AM+FGG / AM) and other independent factors at the 20-year follow-up

|  | **Simple model** | | |  | **Multiple model** | | |
| --- | --- | --- | --- | --- | --- | --- | --- |
|  | **Crude OR** | **95% CI** | **p-value** |  | **Adjusted OR** | **95% CI** | **p-value** |
| **GROUP** |  |  |  |  |  |  |  |
| KT, AM+FGG | 1 |  |  |  | 1 |  |  |
| AM | 7.67 | 1.25 47.0 | **0.028** |  | 19.1 | 1.72 211.9 | **0.016** |
| **Gender** |  |  |  |  |  |  |  |
| Male | 1 |  |  |  | 1 |  |  |
| Female | 1.32 | 0.25 7.10 | 0.746 |  | 1.25 | 0.19 8.38 | 0.819 |
| **AGE** | 0.99 | 0.91 1.10 | 0.971 |  | 0.98 | 0.86 1.11 | 0.747 |
| **SMOKING** |  |  |  |  |  |  |  |
| No | 1 |  |  |  | 1 |  |  |
| Yes | 2.12 | 0.21 21.9 | 0.528 |  | 6.54 | 0.31 138.5 | 0.228 |
| **SPC** |  |  |  |  |  |  |  |
| No | 1 |  |  |  | 1 |  |  |
| Yes | 0.92 | 0.10 8.82 | 0.941 |  | 1.80 | 0.14 23.0 | 0.653 |
| **mPCP** |  |  |  |  |  |  |  |
| No | 1 |  |  |  | 1 |  |  |
| Yes | 3.29 | 0.36 29.9 | 0.292 |  | 4.54 | 0.39 53.2 | 0.228 |

AM, alveolar mucosa; FGG, free gingival graft; KT, keratinized tissue; mPCP, moderate periodontally compromised patients; SPC, supportive periodontal/peri-implant care.

**Supplementary Table 5.** Presence of peri-implant soft-tissue dehiscence (i.e., REC > 1mm). Binary logistic regression (crude and adjusted odds ratio OR; 95%CI) by group (KT / AM) and other independent factors at the 20-year follow-up

|  | **Simple model** | | |  | **Multiple model** | | |
| --- | --- | --- | --- | --- | --- | --- | --- |
|  | **Crude OR** | **95% CI** | **p-value** |  | **Adjusted OR** | **95% CI** | **p-value** |
| **GROUP** |  |  |  |  |  |  |  |
| KT | 1 |  |  |  | 1 |  |  |
| AM | 52.5 | 6.33 435.1 | **<0.001** |  | 81.6 | 8.45 785.9 | **<0.001** |
| **SEX** |  |  |  |  |  |  |  |
| Male | 1 |  |  |  | 1 |  |  |
| Female | 0.69 | 0.26 1.87 | 0.469 |  | 0.51 | 0.12 2.16 | 0.363 |
| **AGE** | 1.01 | 0.96 1.06 | 0.759 |  | 1.00 | 0.93 1.08 | 0.977 |
| **SMOKING** |  |  |  |  |  |  |  |
| No | 1 |  |  |  | 1 |  |  |
| Yes | 0.93 | 0.17 5.01 | 0.936 |  | 1.84 | 0.25 13.5 | 0.551 |
| **SPT** |  |  |  |  |  |  |  |
| No | 1 |  |  |  | 1 |  |  |
| Yes | 0.22 | 0.04 1.11 | 0.067 |  | 0.12 | 0.02 0.91 | **0.04** |
| **mPCP** |  |  |  |  |  |  |  |
| No | 1 |  |  |  | 1 |  |  |
| Yes | 1.44 | 0.52 3.99 | 0.478 |  | 2.26 | 0.46 11.1 | 0.315 |

AM, alveolar mucosa; FGG, free gingival graft; KT, keratinized tissue; mPCP, moderate periodontally compromised patients; SPC, supportive periodontal/peri-implant care.
